# A systematic review of neo-adjuvant radiotherapy in the treatment of breast cancer

**DOI:** 10.3332/ecancer.2021.1175

**Published:** 2021-01-22

**Authors:** Muneer Ahmed, Felix Jozsa, Michael Douek

**Affiliations:** 1 Division of Surgery and interventional Science, University College London, Royal Free Hospital, 9th Floor (East), 2QG, 10 Pond St, London NW3 2PS, UK; 2 Nuffield Department of Surgical Sciences, University of Oxford, Botnar Research Centre, Windmill Road, Oxford OX3 7LD, UK

**Keywords:** neo-adjuvant, radiotherapy, breast cancer, chemo-radiotherapy

## Abstract

**Introduction:**

The use of neo-adjuvant radiotherapy (NRT) has been proven effective at improving cancer related outcome measures, including overall-survival (OS) in the management of solid cancers. However, its utilisation in breast cancer has not been explored to the extent of neo-adjuvant chemotherapy (NAC). The evidence for the application of NRT in breast cancer is evaluated.

**Methods:**

PubMed, Embase and the Cochrane Library databases were searched systematically in August 2020 for studies that addressed the role of NRT in the treatment of breast cancer. Studies were deemed eligible if they reported on objective outcome measurements of OS, disease free-survival (DFS) or pathological complete response (pCR) and attained a satisfactory quality assessment.

**Findings:**

A total of 23 studies reported upon 3,766 patients who had received NRT of which 3,233 also received NAC concurrently (neo-adjuvant chemo-radiotherapy (NCRT)). The pCR values ranged from 14% to 42%, 5-year DFS 61.4% to 81% and 5-year OS 71.6% to 84.2%. Complications were confined to radiation dermatitis with no cases of implant loss reported during breast reconstruction. The application of NRCT alone showed no significant difference in OS or DFS compared to NCRT followed by surgery.

**Interpretation:**

Numbers of patients receiving exclusively NRT is small. However, NCRT is oncologically safe with a low side-effect profile including preceding breast reconstruction. Potential benefits include precise cancer volume targeting, chemosensitisation, elimination of delays in adjuvant therapies and alternatives to chemotherapy in oestrogen receptor positive patients. These factors warrant further exploration within randomised controlled-trials.

## Introduction

The application of both neo-adjuvant and adjuvant therapies in surgical oncology has resulted in a 20% increase in 5-year survival across a range of visceral malignancies over the last 40 years [1]. Adjuvant radiotherapy (RT) utilisation in breast cancer has increased by 29% since 1973 [1] but the role of neo-adjuvant radiotherapy (NRT) has to date been poorly explored. This is despite its extensive clinical application within rectal cancer, in which it has been demonstrated to significantly reduce local recurrence compared to adjuvant RT or surgery alone and improve overall survival (OS) compared to surgery alone [2]. The tumouricidal effects of combining neo-adjuvant radiotherapy and chemotherapy in rectal cancer have also led to a decrease in tumour size, stage of nodal disease and less adverse histological features (lymphovascular invasion and tumour differentiation) [3]. These findings have supported combining neo-adjuvant radiotherapy and chemotherapy in rectal cancer [4]. The enhancement of chemotherapy, avoidance of delay to RT and precise targeting of the cancer *in situ* are all potential benefits of the NRT approach. There is also the future potential to allow for the combining pharmacological interventions with ionising radiation, which more specifically target tumour tissue [5]. The current evidence for the use of NRT as an alternative approach to the management of breast cancer is appraised within this systematic review.

## Methods

### Study selection

A systematic review of the literature was performed using PubMed, Embase and the Cochrane Library databases to identify all original articles published up to August 2020 that evaluated the role of NRT in the management of breast cancer. The search terms used were: Neo-adjuvant radiotherapy AND breast cancer. Studies were restricted to those conducted upon humans and published in the English language. The related articles function was used to broaden the search, and all abstracts, studies and citations obtained were reviewed. References of the acquired articles were also searched by hand. The last search was conducted on 27 August 2020.

### Inclusion criteria

Studies were included if they fulfilled the following eligibility criteria: performance of NRT in primary breast cancer; recorded objective outcome measures in terms of OS, disease free-survival (DFS) or pathological complete response (pCR); attained a satisfactory quality assessment (at least 5 of 7); and were written in the English language.

### Exclusion criteria

Studies that failed to fulfil the inclusion criteria and those in which the outcomes of interest were not reported were excluded. Other exclusion criteria were: full text not available; review article; letter to the editor; editorial report; case report; duplicate publication; published abstracts and articles not in the English language.

### Data extraction

Data were extracted from the selected studies using a data extraction form, which included information on: publication details; study design; number of patients; neo-adjuvant therapy and protocol; type of surgery; adjuvant treatment protocol; follow-up; number of patients achieving pCR; number of local and metastatic recurrences; DFS and OS and complications. The quality of randomised controlled trials (RCTs) was assessed using the ‘risk of bias’ tool from the Cochrane Handbook [6] and cohort studies according to the Strengthening the Reporting of Observational Studies in Epidemiology (STROBE) recommendations [7] and seven items of the STROBE statement were considered relevant for quality evaluation. Two reviewers extracted data from included studies independently. Comparison of the data extraction and quality score was undertaken, and discrepancies were resolved by consensus.

### Statistical analysis

All extracted data were tabulated and presented as means and percentages. Numerators and denominators were provided to address outcomes of included studies.

## Results

**Figure 1.**
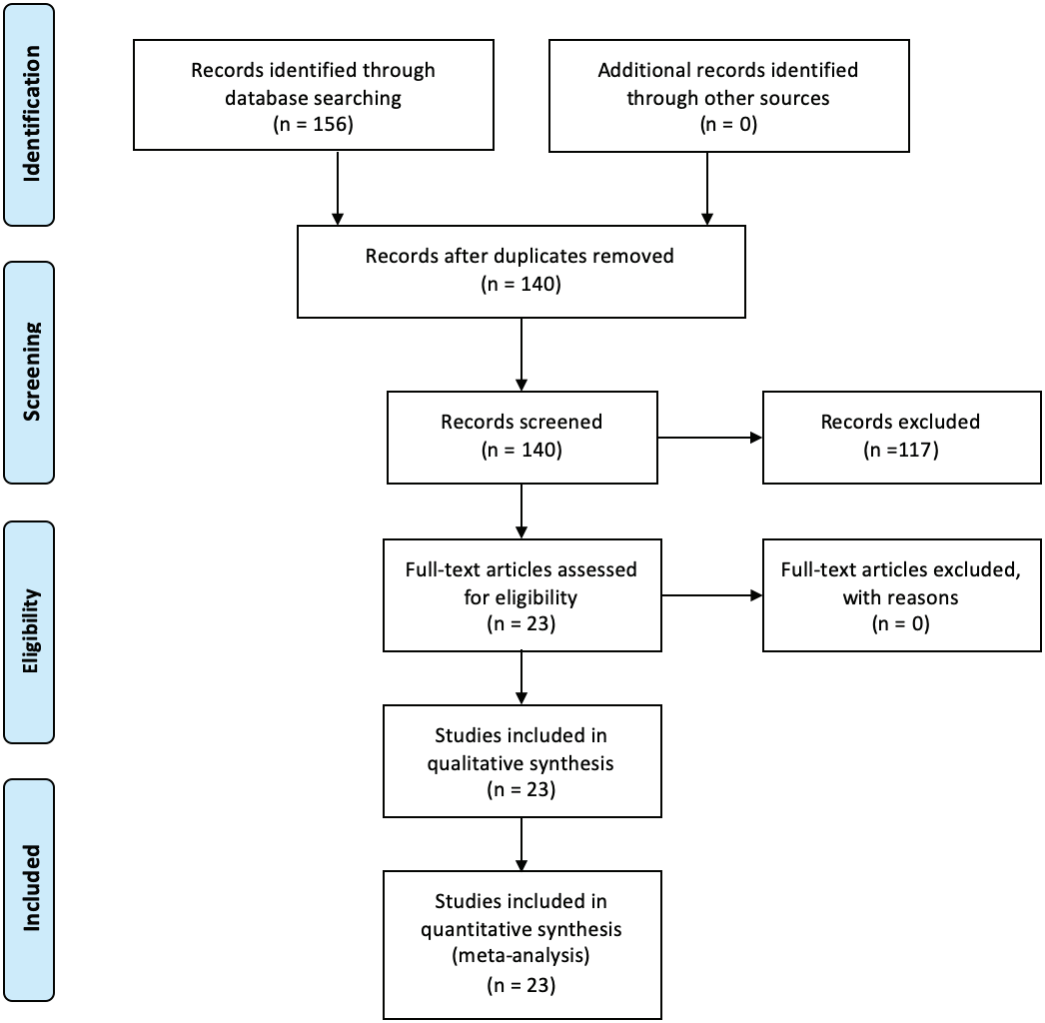
PRISMA flow diagram.

Following the search and screening of published articles (Figure 1), the detailed literature search resulted in 23 studies being critically appraised for this review (Table 1) [8–28].

### Study characteristics

The studies were published between 1994 and 2017 (Table 1). One study was a RCT [23], another cohort-controlled study [22] and the remainder cohort series [8–21] [24–28]. Overall, the studies reported upon 3,766 patients who received NRT, of these only 533 received it alone in three studies [12, 21, 23] with the remaining 18 studies involving patients being treated sequentially with NAC followed by RT. Five studies reported on the use of breast reconstruction, all of which involved pre-operative combination radio and chemotherapy [15, 16, 25, 27]. The NRT protocols administered a radiation dose between 45 and 60 Gy to the whole breast and draining lymph nodes (axillary and extra-axillary including supraclavicular fossa (SCF) and internal mammary nodes (IMNs)) and a boost of between 10 and 15 Gy to the tumour bed. A single study performed targeted volume NRT to the tumour with a total dose of 9.6 Gy [20]. NAC consisted of anthracyclines alone or combined with taxanes and one study using taxanes alone [8]. One study used neo-adjuvant endocrine therapy [17] and another alkylating agent only [23]. Definitive surgery was conducted between 3 and 8 weeks after completion of neo-adjuvant therapies, with breast reconstruction being conducted in five studies [15, 16, 25, 27]. Adjuvant treatments were stated as administered to patients in all trials except two involving breast reconstruction [27, 28]. Follow-up time periods were stated in 12 trials [8, 11, 12, 16–18, 20, 21, 23, 25–28] and ranged between a median value of 14–384 months.

### Study quality

The risk bias tool for the single included RCT is demonstrated in Table 2a. The RCT lacked a power analysis and details regarding blinding of personnel and participants but was overall considered of acceptable quality. The relevant items of the STROBE statement were used for the quality assessment of included cohort studies are shown in Table 2b. The overall STROBE score ranged between 5 and 7. The methodology and reported data of all included studies were considered adequate.

### Results of included studies

The study by Semiglazov *et al* [23] was the only study which randomised patients due to undergo mastectomy to either neo-adjuvant chemotherapy and radiotherapy (NCRT), or to NRT alone. They did not make any comparison with standard adjuvant RT. Two further cohort studies reported outcomes following NRT alone [12, 21]. The authors identified that the pCR rate was 35% and 28% in the NCRT and NRT groups, respectively. The study by Ishitobi *et al* [17] was the only study to use neo-adjuvant endocrine therapy with an aromatase inhibitor in patients undergoing breast-conserving surgery (BCS) and did not identify any cases of pCR. The remaining studies all administered NCRT with a pCR rate reported between 14% and 42% [9, 20]. Five studies [8, 9, 13, 21, 27] evaluated pCR according to tumour receptor status (Table 3) and demonstrated greater pCR rates in oestrogen receptor (ER) negative patients.

DFS at 5-years was reported as 81% versus 71.6% in the NCRT and NRT groups, respectively (*p* < 0.04) [23] and 10-year DFS as 68% versus 67.3% in NCRT and adjuvant treatment groups [22] when directly compared in studies. The OS at 5-years in the NCRT versus NRT study [23] was 86% and 78%, respectively, and 69% versus 65% in the NCRT and adjuvant treatment groups [22]. When NCRT was not followed by definitive surgery, the 10-year DFS was 52% and 61% (*p* = 0.73) and OS 77% and 79% for no surgery and surgery, respectively [14]. In the other studies with patients undergoing NRT alone, Riet *et al* [21] reported a pCR rate of 10% with 30% OS and DFS at 25-year follow-up. Calitchi *et al* [12] reported a 47% and 55% DFS and OS at 15-year follow-up.

In the cohort studies reporting 10-year follow-up after neo-adjuvant radiochemotherapy (NRCT), the DFS was reported between 52.6% and 68%, with OS at the same time point being between 63% and 69% [18, 22, 24, 26]. Two further studies, which recorded 5-year DFS in NCRT cohort’s reported figures between 61.4% and 76.9% and OS between 71.6% and 84.2% [8, 9]. A single study of 15-year DFS in a NCRT cohort reported a figure of 47% and OS of 55% [12]. The study by Ishitobi *et al* [17] of neo-adjuvant endocrine therapy demonstrated 24 out of 25 patients alive and disease-free at a median of 18 months follow-up. Nardone and Pazos *et al* [20, 28] reported 19 of 21 and 18 out of 22 patients disease-free at median follow-up of 30 months. The authors also reported all 21 and 18 out of 22 patients alive at follow-up [20, 28].

The incidence of loco-regional and distant metastatic disease is shown in Table 4. The study with the longest median follow-up after preoperative NRT alone of 384 months showed an 8% local recurrence rate [21]. This was followed by 15 year follow-up of a NRT cohort which reported local recurrence and metastatic rates of 12% and 36%, respectively [12]. Lerouge *et al* [18] reported a 9% local recurrence rate and 8.3% of metastatic cases after a 140 month median follow-up of NCRT. Bollet *et al* [11] reported a median follow-up of 84 months, with 12% and 22% local recurrences and metastatic cases, respectively.

Studies with follow-up of up to 60 months demonstrated lower rates of between 0.8% and 10% for local recurrence and 3.5% and 23% for cases of metastases [8, 9, 16, 17, 20, 25, 28]. The study by Daveau *et al* [14] demonstrated no significant difference in metastatic cases between NCRT followed by surgery or no surgery. Whilst this was replicated in the local recurrence rate, there was a trend towards better local control in the surgery groups (16.9% versus 32%).

Five studies reported the use of breast reconstruction in a total of 264 patients following NRCT and primary surgery [10, 15, 16, 25, 27]. Of these, 232 patients (87.8%) underwent autologous reconstruction, and the remaining 32 patients’ reconstructions involved prosthetic implants. All patients that received implants came from the same study [27], in which they were used to augment latissimus dorsi (LD) flaps. In those patients receiving autologous reconstruction, 87 had a transverse rectus abdominis (TRAM) flap, 176 had an LD flap and 1 patient had a combination of TRAM/LD flaps. The pCR in the breast reconstruction studies ranged from 21.7% to 58.2% [10, 27]. Follow-up was reported in two of these studies at median of between 24 and 42 months [16, 27]. Complications relating to breast reconstruction included cellulitis, partial flap necrosis and fat necrosis. Ho *et al* [16] reported donor site complications in 20% of patients and a single case of flap necrosis requiring debridement. The remaining studies all demonstrated skin complications, which settled with conservative management, including the study by Zinzindohoue *et al* [27]—that included 38.5% of patients undergoing implant based reconstruction—reporting five cases of skin necrosis, which healed at 2 months with surgical revision and dressings.

Complications were mainly distributed between neutropenia and anaemia and skin changes (Table 4). The study by Alvarado-Miranda *et al* [9] reported radiation dermatitis in 22.4% of patients compared to only 7.7% in the study by Semiglazov *et al* [23]. There were no cases of neutropenic sepsis reported.

## Discussion

NAC has become widely applied in the treatment of locally advanced breast cancer but the performance of NRT has not. This is in contrast to other malignancies in which it has become established as a standard of care, with improved OS [2]. The studies within this review demonstrate that the administration of NRT—in comparable doses to adjuvant RT—is well tolerated in breast surgical oncological procedures [9, 14, 23] and when combined with breast reconstruction [27]. The combination of NCRT when directly compared against NRT alone demonstrated superior pCR rates, DFS and OS at 5 years—although only DFS reached statistical significance [23]. This would support the combined, enhanced tumouricidal effects of NCRT compared to NRT alone, which have already been demonstrated in rectal cancer [3]. These tumouricidal effects were not replicated with the application of neo-adjuvant endocrine therapy, where no cases of pCR were reported [17]. A pCR was significantly associated with hormone receptor negative compared to positive cancers on univariate analysis (*p* < 0.002) [9]. This is demonstrated by the increased rates of pCR—as would be expected—in those patients that are ER negative compared to positive (Table 4a). This supports the theory of chemosensitisation of certain cell lines by administration of concurrent RT, preventing the activation of pro-survival transcription factors and the MDR-1 gene [20]. This clearly reiterates the importance of molecular phenotype of tumours with respect to their hormone receptor and HER2 status in determining their likely response to neo-adjuvant therapy. From the poor pCR results of neo-adjuvant endocrine therapy combined with NRT, it is suggestive that there is a lack of a hormone sensitising effect by concurrent RT [17]. The Surveillance, Epidemiology, and End Results (SEER) database analysis of 250,195 women with early breast cancer who underwent NRT (2,554) and adjuvant RT (247,641) demonstrated that NRT resulted in a lower hazard ratio for a second primary tumour at any location among ER positive patients compared to adjuvant RT (HR 0.64, 95% CI 0.55–0.75; *p* < 0.0001) and in those undergoing NRT and mastectomy compared with those who received adjuvant RT (HR 0.48; 95% CI 0.26–0.87; *p* = 0.02) [29]. Therefore, an important role of NRT combined with endocrine therapy may be to allow greater time for maximal tumour regression and avoidance of chemotherapy in a certain subset of patients, with improved outcomes.

The presence of a clearly visualised target pre-operatively for the administration of NRT is a clear advantage absent from adjuvant RT. This allows the application of whole breast RT with a boost to the precise target site or targeted RT to the tumour and surrounding normal tissue, without fear of missing the tumour bed. This strategy avoids the need to estimate the approximate position of the original tumour—as standardly directed by surgically placed titanium clips intra-operatively. This is of increasing clinical importance given the wider use of oncoplastic surgery, challenging subsequent adjuvant RT planning. The evaluation of NCRT for exclusive management without surgery demonstrated that DFS, OS, occurrence of metastatic and local disease were not significantly different [14]. These findings were in spite of only 41% of the non-surgical group undergoing a complete radiological response to NCRT [14]. This demonstrates the potential for NCRT to be performed as standalone therapy without surgery. The non-significant trend towards greater local relapse in the NCRT group was demonstrated on univariate analysis to be related to larger tumour size and younger age [14]. Therefore, careful selection of patients to avoid this subset of risk factors and consideration of molecular phenotyping could make this surgical-free treatment option feasible.

The impact of timing of administration of RT in breast reconstruction varies between autologous and implant based. The Mastectomy Reconstruction Outcome Consortium [30] prospective cohort of patients undergoing autologous reconstruction demonstrated that no differences in complications were identified in patients receiving chest wall RT between delayed and immediate breast reconstruction. An insurance-claims based series of nearly 5,000 patients [31] demonstrated that RT use in implant-based reconstruction is associated with an 11 times greater likelihood of failure compared with irradiated autologous reconstruction. However, with the former, delayed reconstruction after RT is associated with the highest probability of implant failure. In the five studies [10, 15, 16, 25, 27] using breast reconstruction, all involved autologous tissue and a single study autologous tissue with implants [27]. Whilst skin related complications were reported, no cases of flap failure or implant loss were reported and all cases settled with conservative management or minimal debridement. This would suggest that NRT can be applied to the reconstructive setting and avoid the problems of adjuvant RT with respect to its delayed administration should a surgical complication arise.

Within this review, it has been shown that when NCRT is compared to the standard of adjuvant treatment, no significant difference in DFS or OS was identified at 10 years [22]. The DFS, OS and recurrence rates of included studies [8, 9, 14, 22, 23] recording these outcomes are comparable to the gold standard of adjuvant treatment—as recorded at meta-analysis [32]. This is supported by the SEER database, which suggested that the ER positive patient population could experience significant benefits in reducing disease recurrence [29]. Clearly, there is heterogeneity amongst the studies regarding their NCRT protocols and the administration of adjuvant therapies as well as inclusion of only a single RCT—of NRT versus NRCT. This illustrates that currently RT is not being utilised within neo-adjuvant treatments. This review is inherently limited due to the lack of available evidence but highlights that the addition of NRT to NAC has a low side effect profile—including with breast reconstruction—and is oncologically safe.

## Conclusion

The application of NRT in the treatment of breast cancer patients can streamline oncological treatment, provide chemosensitisation to enhance pCR prior to definitive surgery and provide treatment alternatives to ER positive patients who are less likely to respond to chemotherapy. Indeed, there is even potential that in a carefully selected subgroup of patients according to their histopathological and molecular features, the need for surgical intervention may be obviated. The potential benefits of NCRT (with or without biological agents) now warrant further exploration within prospective, RCTs to evaluate their potential benefits, in addition to that of NRT versus the standard of adjuvant RT.

## Conflicts of interest statement

The authors have no disclosures to make concerning financial or personal relationships that could inappropriately influence their work.

## Funding declaration

No funding was received for this study.

## Figures and Tables

**Table 1.** Characteristics of included studies.

(a) Involving preoperative RT and CT									
Study and type	Total no. of pts	Neo-adjuvant therapy		Neo-adjuvant protocol		Surgery (BCS versus Mx) and timingß	Adjuvant treatment protocol		Median follow-up (months)
		RT	NAC	RT	NAC		RT	AC	
Touboul *et al* [26]	97 (IIIA–IV)	97	97	Whole breast, chest wall, regional lymph nodes irradiated (total 45 Gy in 23 fractions), 3 weeks after NAC	Doxorubicin day 1 (45 mg/m^2^) plus vincristine day 2 (1.5 mg/m^2^) plus 5-fluorouracil (500 mg/m^2^) and cyclophosphamide (300 mg/m^2^) day 2, 3, 4. Four cycles, repeated every 28 days	27:37 no surgery (*n* =33) 4 weeks after NAC	30 Gy boost in 15 fractions in those patients whose primary tumour had disappeared (*n* = 33)	Anthracycline (30 mg/m^2^)	93
Colleoni *et al* [13]	32 (T2–T4)	29	32	Whole breast irradiation (50 Gy) and boost to tumour nodule (10 Gy), 3–4 weeks following NAC	Doxorubicin (60 mg/m^2^) and cyclophosphamide (600 mg/m^2^) for three courses 21 days apart	25:7 ANC performed in all patients	NA	NA	NA
Skinner *et al* [33]	36 (IIB–IV)	30	30	Total 50 Gy in 25 fractions, beginning on day 15 of 5-FU,	5-flurouracil (200 mg/m^2^) for eight consecutive weeks	All patients received mastectomy within 4–6 weeks	NA	Adriamycin (*n* = 28); tamoxifen (*n* = 2)	22 (8–40)
Lerouge *et al* [18]	120 (IIIA–IIIC)	120	120	Whole breast and regional lymph nodes (45 Gy in 23 fractions over 31 days), 3 weeks after the fourth cycle of NAC	Four cycles of either doxorubicin day 1 (45 mg/m^2^) plus vincristine day 2 (1.5 mg/m^2^) plus 5-fluorouracil (500 mg/m^2^) and cyclophosphamide (300 mg/m^2^) day 2, 3, 4 (*n* = 94); or theprubicin day 1 (40 mg/m^2^) plus vindesine day 2 (2 mg/m^2^) plus 5-fluorouracil (500 mg/m^2^) and cyclophosphamide (300 mg/m^2^) day 2, 3, 4 and 5 (*n* = 16). Cycles repeated every 21 days. 4 weeks after RT, a fifth cycle of CT was given	71:49 8 weeks following completion of RT	NA	vincristine (1.5 mg/m^2^) on Day 1; 5-fluorouracil 500 mg/m^2^) and cyclophosphamide 300 mg/m i.v. on Days 2, 3 and 4 (*n* = 94). Or 6 monthly cycles of vinorelbine (25 mg/m^2^) on Days 1 and 5, 5-fluorouracil (350 mg/m^2^) from Day 1 to Day 5, and leucovorin 250 (mg/m^2^) on Days 2 and 4 (*n* = 26)	140
Chakravarthy *et al* [34]	34 (IIA–IIIB)	30	34	Whole breast (total 4,680 cGy in 28 fractions) and regional nodes irradiated (total 4,500 cGy in 25fractions)	Paclitaxel (175 mg/m^2^) every 3 weeks for three cycles, followed by paclitaxel (30 mg/m^2^) twice-weekly	16:21 3–4 weeks following completion of RT	NA	Four cycles of doxorubicin/cyclophosphamide (n = 28)	23 (1–46)
Shanta *et al* [24]	1,117 (IIB–IIIB)	1,117	1,117	Tumour dose (total 4,000 cGy in 20 fractions), and additional dose to posterior axillary fields RT began on day 2 following start of NAC	Two regimens (not randomised) 1. Cyclophosphamide (600 mg/m^2^), 5-fluorouracil (600 mg/m^2^) and methotrexate (40 mg/m^2^). (*n* = 954)2. Cyclophosphamide (600 mg/m^2^), 5-fluorouracil (600 mg/m^2^) and adriamycin (75 mg/m^2^) or epirubicin (90 mg/m^2^). (*n* = 163) Both regimens given three courses at 3-weekly intervals	NS ‘usually mastectomy’ 3 weeks after NAC	Internal mammary RT (total 4,000 cGy in 20 fractions)	All patients given 4th CT cycle 8–12 days postoperatively	NS (varied)
Alvarado-Miranda *et al* –[9] Cohort series	112 (IIB–IIIB)	112	112	Whole breast and nodal areas following NAC; 60 Gy divided into 50 Gy in 5 weeks plus boost 10 Gy in 1 week to palpable disease	5-flurouracil (500 mg/m^2^), Adriamycin (50 mg/m2), cyclophosphamide (500 mg/m^2^) (FAC) or adriamycin (50 mg/m^2^) and cyclophosphamide (500 mg/m^2^) (AC) in 4, 21 day courses. During RT, mitomycin c (5 mg/m^2^), 5-flurouracil (500 mg/m^2^) and dexamethasone (16 mg) or cisplatin (30 mg/m^2^), gemcitabine (100 mg/m^2^) and dexamethasone (16 mg) (six cycles)	6-8 weeks; (0:112) (ANC performed in all patients)	NA	FAC or AC; two additional courses and endocrine therapy if ER positive	43^h^
Adams *et al* [8] Cohort series	105 (IIB–IIIC)	105	105	Breast, axillary and SCF nodes (weeks 2–7), 1.8 Gy per fraction to total dose of 45 Gy + boost of 14 Gy at 2 Gy per fraction to originally palpable tumour.	30 mg/m^2^ paclitaxel bd, 10–12 weeks. Trastuzumab (2 mg/kg) if HER-2 positive	4 weeks (all patients received ANC)	NA	Combination chemotherapy and endocrine therapy (ER positive)	60
Roth *et al* [22] Cohort-controlled series	644^c^ (IIA–IIIC)	315	315	50 Gy whole breast and SCF, 5 × 2 Gy/week	Epirubicin and cyclophospham-ide (four cycles) or adriamycin and cyclophospham-ide (four cycles) or cyclophospham-ide, methotrexate, 5-fluorouracil (three or six cycles) or epirubicin and cyclophospham-ide (six cycles)	(160:155)	50 Gy plus 10 Gy boost in BCS^c^	As NAC but with taxane regime also included^c^ Endocrine therapy if ER positive	NS
Daveau *et al* [14] Cohort controlled series	165 (T2–3 ≤ 7 cm)	165	165	Whole breast and loco-regional nodal areas, 45 Gy + boost of 10–15 Gy	Adriamycin (25 mg/m^2^), cyclophospham-ide (500 mg/m^2^) and 5-fluorouracil (500 mg/m^2^) or docetaxel (75 mg/m^2^) and Adriamycin (50 mg/m^2^); 4 weekly for six cycles	65^a^ (53:12) (ANC in all patients)	NA	Endocrine therapy for ER positive patients. Chemo-therapy for heavy axillary involvement	NS
Bollet *et al* [11]	59 (T2–3)	59	59	Whole breast irradiation to 50 Gy in 5 weeks. Internal mammary chain and supra/infra-clavicular nodes irradiated to 46 Gt in 4.6 weeks	5-flurouracil (500 mg/m^2^/d) over five consecutive days, and vinorelbine (25 mg/m^2^) on day 1 and 6. Repeated every 3 weeks for a total of four courses	41:18 Minimum 6 weeks ANC performed in all patients	RT boost in young patients or margins at risk (*n* = 37)	None (*n* = 7), or 5-FU, epirubicin and cyclophosphamide in absence of complete pathological response (*n* = 10) and/or hormone therapy where indicated (hormone therapy alone *n* = 12, hormone and chemotherapy *n* = 30)	84 (60–96)
Ishitobi *et al* [17] Cohort series	25 (T ≥ 3 cm, N0–2)	25	25^b^	50 Gy in 25 fractions to breast and SCF (if node positive)	Anastrazole 1 mg/day for 24 weeks	25 (25:0) and ANC or SNB	NA	Adjuvant anastrazole (all patients); trastuzamab if HER-2 positive and chemo-therapy selectively	14 (14–29)
Matuschek *et al* [19] Cohort series	315 (T1–T4/N0–N1)	315	315	50 Gy whole breast and SCF, 5 × 2 Gy/week	Epirubicin and cyclophosphamide (four cycles) or adriamycin and cyclophosphamide (four cycles) or cyclophosphamide, methotrexate, 5-fluorouracil (four cycles) or epirubicin and cyclophospha-mide (six cycles)	160:155	NA	Endocrine therapy if ER positive	NS
Nardone *et al* [20] Cohort series	21^g^	21	21	9.6 Gy (6 × 21 day cycles) using a clinically targeted volume	Liposomal anthracycline (50 mg/mq) and docetaxel (75 mg/mq) (six cycles)	(18:3) 3 weeks	50.4 Gy to whole breast or chest wall	AC and endocrine therapy if ER positive	31
Pazos *et al* [28] Cohort series	22 (T1–4/N0–2)	22	22	Whole breast and SCF; 50.4 Gy (5 × 1.8 Gy/week)	Epirubicin (90 mg/m2) and cyclophosphamide (600 mg/m^2^) (four cycles) followed by paclitaxel (80 mg/m2) (12 cycles)	(22:0)^e^ 47 days (26–162 days)^∑^	NA	NS	30

(b) Involving preoperative RT only									
Study and type	Total no. of pts	Neo- adjuvant therapy		Neo-adjuvant protocol		Surgery (BCS versus Mx) and timing ß	Adjuvant treatment protocol		Median follow-up (months)
		RT	NAC	RT	NAC		RT	AC	
Semiglazov *et al* [23] RC	271 (IIB–IIIA)	271^d^	137	60 Gy, single daily dose of 2 Gy; SCF 40 Gy, single daily dose of 2 Gy	Thiotepa (120 mg total dose), methotrexate 40 mg/m^2^, 5-fluorouracil 500 mg/m^2^, 1–2 cycles	(0:271) 3–4 weeks	NA	4–6 cycles of AC	53
Calitchi *et al* [12] Cohort series	75 (T2–3)	75	0	45 Gy (5 weeks) to whole breast, lower axillary nodes (including 15 GY boost to IMNs)	-	(75:0)	NA	AC and endocrine therapy if ER positive	120
Riet *et al* [21]	187 (T2–T4)	187	0	Whole breast irradiation, SCF and axilla (total 45 Gy in 18 fractions)	-	All patients received mastectomy plus ANC At least 4 weeks following completion of RT	NA	*n* = 58 (NS)	384 (264–420)

(c) Involving preoperative RT and/or CT, with breast reconstruction									
Study and type	Total no. of pts	Neo-adjuvant therapy		Neo-adjuvant protocol		Surgery (BCS versus Mx) and timing ß	Adjuvant treatment protocol		Median follow-up (months)
		RT	NAC	RT	NAC		RT	AC	
Aryus *et al* [10]	55 (I–IV)	55	55	Total 50 Gy with boost to tumour	Cyclophosphamide (600 mg/m^2^), fluorouracil (600 mg/m^2^) and methotrexate (40 mg/m^2^); or cyclophosphamide (600 mg/m^2^) and epirubicin (90 mg/m^2^)	Median 27 (11–21) weeks after NAC/RT	NS	NS	NS
	Seven patients had mastectomy + immediate TRAM recon (presumed pedicled). Twenty-eight underwent tumourectomy + LD reconstruction. No flaps were lost in the chemo-RT group, but no details given regarding other surgical complications								
Skinner *et al* [25]	27 (IIB–IV)	27	27	Total 45 Gy (1.8 Gy/fraction over 5 weeks)	2 weeks of paclitaxel 60 mg/m^2^, after completion of RT	All patients received mastectomy Within 4–6 weeks	NA	Four cycles of adriamycin-based polychemotherapy; 5-year course of tamoxifen for ER-positive patients	NS
	Two patients underwent immediate TRAM reconstruction (presumed pedicled, but not stated)—one developed early partial flap failure requiring revision; the other wound problems and delayed healing								
Gerlach *et al* [15]	134 (I–IV)	134	134	Total 50 Gy in 2 Gy fractions. Internal mammary lymph nodes irradiated (*n* = 9)	Epirubicin/cyclophosphamide plus cyclophosphamide/methotrexate/fluorouracil (*n* = 50); Epirubicin/cyclophosphamide only (*n* = 59); other (*n* = 25)	74:60 Median 8 (4–24) weeks delay after NAC/RT	NS	NS	NS
	22/134 underwent mastectomy + immediate TRAM reconstruction (presumed pedicled, but not stated) while 60/134 underwent tumourectomy + LD reconstruction. No details were given regarding surgical complications								
Ho *et al* [16]	30 (IIIA–IIIC)	30	30	Breast and chest wall. Total 50 Gy in 25 fractions (*n* = 18); total 42.5 Gy in 16 fractions (*n* = 12). Median 6.25 Gy boost dose given to *n* = 26 patients	Adriamycin and cyclophosphamide (*n* = 1); fluorouracil, Adriamycin, cyclophosphamide (*n s=* 6); cyclophosphamide, epirubicin, fluorouracil (*n* = 4); Adriamycin and taxane (*n* = 15); fluorouracil, epirubicin, cyclophosphamide (*n* = 2)	All patients received skin-sparing mastectomy. Median delay 6.9 (2.7–12.9) weeks following RT	NA	Tamoxifen where indicated	42 (12–113)
	Pedicled TRAMs (*n* = 24), LD flaps (*n* = 5) and a combination of TRAM and LD flaps (*n* = 1). Local complications in *n* = 11 (37%) included mastectomy flap necrosis (*n* = 3), partial flap necrosis (*n* = 1), fat necrosis (*n* = 1) and flap fibrosis (*n* = 1). Donor site complications were reported in 20%								
Zinzindohoue *et al* [27] Cohort series	83^f^	83	83	50 Gy to breast, axillary, IMN or SCF nodes	Anthracyclines and taxane regimens	(0:83)^e^ 6–8 weeks	NA	NS	24
	Reconstruction was performed using autologous LD flap with or without prosthesis. Prostheses were used for IBR in 32 patients (mean volume 256 ± 73 mm^3^). Five patients had necrosis, all recovered without revision surgery								

NS, Not stated; NA, Non-applicable; BCS, Breast conserving surgery; Mx, Mastectomy; RT, Radiotherapy; NAC, Neo-adjuvant chemotherapy; ANC, Axillary node clearance; ER, Oestrogen receptor; RCT, Randomised controlled trial; IMNs, Internal mammary nodes; SCF, Supraclavicular fossa; (), breast cancer staging; AC, Adriamycin and cyclophosphamide; FAC, 5-fluorouracil, adriamycin and cyclophosphamide; no., Number; pts, Patients; ß, Timing after completion of neo-adjuvant therapies; ∑, Median with range

a Patients undergoing surgery after neo-adjuvant RT (reminder treated conservatively)

b Neo-adjuvant endocrine therapy

c Includes 329 patients in adjuvant only treatment arm

d Includes patients receiving neo-adjuvant RT + NAC and neo-adjuvant RT alone

e Includes breast reconstructions

f Patients necessitating mastectomy

g Invasive breast cancer

h Mean

**Table 2.** Outcomes of included studies.

(a) Involving preoperative RT and CT							
Study	Number of patients achieving pCR	Number of local recurrences	Number of metastatic cases	DFS (%)	OS (%)	Number of complications	
						Skin-related	Other
Touboul *et al* [26]	41 (complete remission)	5	5	61^b^	69^b^	NS	N+V (*n* = 29); hair loss (*n* = 90)
Colleoni *et al* [13]	2	NS	NS	NS	NS	NS	5
Skinner *et al* [33]	6	NS	3	83; ^f^27	NS	9	9
Lerouge *et al* [18]	8	11	10	60^a^	^g^66.5	NS	NS
Chakravarthy *et al* [34]	13	NS	NS	NS	NS	1	10
Shanta *et al* [24]	NS	Local *n* = 17; locoregional *n* = 16; regional only *n* = 46	NS	52.6^b^; 41.4^c^	63.9^b^; 58.4^c^	NS	NS
Alvarado- Miranda *et al* [9]	47 (65)	1	4	76.9 (95% CI, 68.2–84.7)	84.2 (95% CI, 75–93)	25	6
Adams *et al* [8]	36	5	24	61.4 (95% CI, 50.1–70.8)	71.6 (95% CI, 60.5–80.1)	NS	NS
Roth *et al* [22]	116 (61)	NS	NS	68^b^—NCRT group 67.3^b^—Adjuvant group	68.6^b^—NCRT group 65^b^—Adjuvant group	NS	NS
Daveau *et al* [14]	41^e^—no surgery 8 (19^e^)— surgery	32—no surgery 11—surgery	21 (27)^b^—no surgery 14 (26)^b^—surgery	65 (52)^b^—no surgery 72 (61)^b^—surgery	91 (77)^b^—no surgery 82 (79)^b^—surgery	3—no surgery	0
Bollet *et al* [11]	16	7	13	83^a^	88^a^	NS	Grade 3 (*n* = 4); grade 2 (*n* = 16); grade 1 (*n* = 18)
Ishitobi *et al* [17]	0	1	1	^f^24	^g^24	2	1
Matuschek *et al* [19]	116	NS	NS	NS	NS	NS	NS
Nardone *et al* [20]	3	0	2	19**	21^g^	1	NS
Pazos *et al* [28]	5	2	2	^f^18	^g^18	NS	NS

(b) Included studies involving preoperative RT alone							
Study	Number of patients achieving pCR	Number of local recurrences	Number of metastatic cases	DFS (%)	OS (%)	Number of complications	
						Skin-related	Other
Semiglazov *et al* [23]	17—NCRT group 8—NRT group	NS	NS	81—NCRT group 71.6—NRT group	86.1 —NCRT group 78.3—NRT group	9—NCRT 12—NRT	NS
Calitchi *et al* [12]	NS	9	27	47^c^	55^c^	2	NS
Riet *et al* [21]	18	15	NS	30^d^	30^d^	8	29

(c) Included studies involving preoperative RT and/or CT, with breast reconstruction							
Study	Number of patients achieving pCR	Number of local recurrences	Number of metastatic cases	DFS (%)	OS (%)	Number of complications	
						Skin-related	Other
Aryus *et al* [10]	32	NS	NS	NS	NS	NS	NS
Skinner *et al* [25]	7	NS	NS	NS	NS	7	1
Gerlach *et al* [15]	56	NS	NS	Median 25 (6–36) months tumour free survival	19 (2–64) months OS time	NS	NS
Ho *et al* [16]	NS	3	23% of 30 patients had distant relapse	68^a^	NS	7	6
Zinzindohoue *et al* [27]	18	NS	NS	NS	NS	5	1

NS, Not stated; pCR, Pathological complete response in breast; (), Pathological complete response in axillary nodes (pCRA); NCRT, Neo-adjuvant chemotherapy and radiotherapy; NRT, Neo-adjuvant radiotherapy;

a 5-year follow-up values

b 10-year follow-up values

c 15-year follow-up values

d 25-year follow-up values

e Based upon imaging

f Number of patients free of disease at median follow-up

g Number of patients alive at median follow-up

^h^Calculated mean of OS from pCR and non-pCR groups

^i^As a proportion of 50 patients with upfront decision for mastectomy only

**Table 3.** Methodological characteristics and quality assessment of included studies.

(a) Cohort studies involving preoperative RT and/or CT						
Study	Study objectives	Clear inclusion criteria	Standardised treatment technique	Standardised histopathology assessment	Patient follow-up reported	Withdrawals from study reported
Touboul *et al* [26]	Yes	Yes	Yes	Yes	Yes	Yes
Colleoni *et al* [13]	Yes	Yes	Yes	NS	NS	NS
Skinner *et al* [33]	Yes	Yes	Yes	NS	Yes	Yes
Lerouge *et al* [18]	Yes	Yes	Yes	Yes	Yes	Yes
Chakravarthy *et al* [34]	Yes	Yes	Yes	Yes	Yes	Yes
Shanta *et al* [24]	Yes	Yes	Yes	NS	Yes	Yes
Alvarado-Miranda *et al* [9]	Yes	Yes	Yes	Yes	Yes	Yes
Adams *et al* [8]	Yes	Yes	Yes	Yes	Yes	Yes
Roth *et al* [22]	Yes	Yes	Yes	Yes	NS	Yes
Daveau *et al* [14]	Yes	Yes	Yes	NS	NS	Yes
Bollet *et al* [11]	Yes	Yes	Yes	Yes	Yes	Yes
Ishitobi *et al* [17]	Yes	Yes	Yes	Yes	NS	Yes
Matuschek *et al* [19]	Yes	Yes	Yes	Yes	NS	Yes
Nardone *et al* [20]	Yes	Yes	Yes	Yes	Yes	Yes
Pazos *et al* [28]	Yes	Yes	Yes	NS	Yes	Yes

(b) Cohort studies involving preoperative RT only						
			
Study	Study objectives	Clear inclusion criteria	Standardised treatment technique	Standardised histopathology assessment	Patient follow-up reported	Withdrawals from study reported
Calitchi *et al* [12]	Yes	Yes	Yes	No	Yes	Yes
Riet *et al* [21]	Yes	Yes	Yes	Yes	Yes	Yes

(c) Cohort studies involving preoperative RT and/or CT with breast reconstruction						
Study	Study objectives	Clear inclusion criteria	Standardised treatment technique	Standardised histopathology assessment	Patient follow-up reported	Withdrawals from study reported
Aryus *et al* [10]	Yes	Yes	Yes	Yes	Yes	Yes
Skinner *et al* [25]	Yes	Yes	Yes	Yes	NS	Yes
Gerlach *et al* [15]	Yes	Yes	Yes	Yes	NS	Yes
Ho *et al* [16]	Yes	Yes	Yes	NS	Yes	Yes
Zinzindohoue *et al* [27]	Yes	Yes	Yes	Yes	Yes	Yes

(d) Randomised control trials							
Study	Power analysis	Adequate sequence generation	Allocation concealment	Blinding (participants and personnel and all outcomes)	Incomplete data addressed	Free of selective reporting	Free of other bias
Semiglazov *et al* [23]	No	Yes	Yes	NS	Yes	Yes	Yes
NS, Not stated							

**Table 4.** Outcome of included studies reporting data by tumour phenotype. (a) Pathological complete response reported by tumour phenotype

Study	pCR (%)			
	ER+	ER−	HER2+	Triple negative
Riet *et al* [21]				26
Zinzindohoue *et al* [27]	44.5	55.5	11	
Colleoni *et al* [13]	38	44		
Alvarado-Miranda *et al* [9]	54 [45–63]	81 [74–88]		
Adams *et al* [8]	52^c^	50^c^	32^c^	

(b) Locoregional recurrence and distant DFS reported by tumour phenotype								
Study	Locoregional recurrence rate (%)				Distant DFS (%)			
	ER+	ER−	Her2+	Triple negative	ER+	ER−	Her2+	Triple negative
Bollet *et al* [11]	93^a^ [85–100]	80^a^ [61–100]	75^a^ [50–-100]	91^a^ [83–100]	88a [78–-98]	69^a^ [49–96]	75^a^ [50–100]	75^a^ [54–100]
Lerouge *et al* [18]					69.9^b^ (+/−8)	64.3^b^ (+/−11.8)		
After NRT alone					68.4^b^ (+/−-10.7)	51.6^b^ (+/−14.5)		
Alvarado-Miranda *et al* [9]					93^a^ (+/−3)	83^a^ (+/−4)		

^a^5-year^b^10-year^c^Includes complete and partial pathological response
